# The IMPACT study: A clustered randomized controlled trial to assess the effect of a referral algorithm for axial spondyloarthritis

**DOI:** 10.1371/journal.pone.0227025

**Published:** 2020-01-28

**Authors:** Maha Jamal, Amber M. Korver, Martijn Kuijper, Deirisa Lopes Barreto, Cathelijne W. Y. Appels, Anneke P. L. Spoorenberg, Bart W. Koes, Johanna M. W. Hazes, Lonneke van Hoeven, Angelique E. A. M. Weel

**Affiliations:** 1 Department of Rheumatology and Clinical Immunology, Maasstad Hospital, Rotterdam, The Netherlands; 2 Department of Rheumatology, Erasmus MC University Medical Centre, Rotterdam, The Netherlands; 3 Department of Rheumatology, Amphia Hospital, Breda, The Netherlands; 4 Department of Rheumatology and Clinical Immunology, University Medical Centre Groningen, Groningen, The Netherlands; 5 Department of General Practice, Erasmus MC University Medical Centre, Rotterdam, The Netherlands; VU University Medical Center, NETHERLANDS

## Abstract

**Background:**

A substantial number of patients with chronic low back pain (CLBP) have axial spondyloarthritis (axSpA), but early recognition of these patients is difficult for general practitioners (GPs). The Case Finding Axial Spondyloarthritis (CaFaSpA) referral strategy has shown to be able to identify patients with CLBP at risk for axSpA, but its impact on clinical daily practice is yet unknown.

**Objective:**

To assess the effect of the CaFaSpA referral strategy on pain caused by disability in primary care patients with CLBP.

**Methods:**

Within this clustered randomized controlled trial 93 general practices were randomized to either the CaFaSpA referral model (intervention) or usual primary care (control). In each group primary care patients between 18 and 45 years with CLBP were included. The primary outcome was disability caused by CLBP, measured with the Roland Morris Disability Questionnaire (RMDQ) at baseline and four months. Secondary outcome was the frequency of new axSpA diagnosis. Descriptive analyses were performed, and a linear mixed-effects model was used.

**Results:**

In total 679 CLBP patients were included of which 333 patients were allocated to the intervention group and 346 to the control group. Sixty-four percent were female and mean age was 36.2 years. The mean RMDQ score at baseline was 8.39 in the intervention group and 8.61 in the control group. At four months mean RMDQ score was 7.65 in the intervention group and 8.15 in the control group. This difference was not statistically significant (p = 0.50). Six (8%) out of the 75 finally referred patients, were diagnosed with axSpA by their rheumatologist.

**Conclusions:**

The CaFaSpA referral strategy for axSpA did not have an effect on disability after four months caused by CLBP. However, the strategy is able to detect the axSpA patient within the large CLBP population sufficiently.

Trial registration number: NCT01944163, Clinicaltrials.gov.

## Introduction

Axial spondyloarthritis (axSpA) is a chronic disabling rheumatic disease for which the leading symptom is chronic low back pain (CLBP). [1] The prevalence of axSpA among CLBP patients varies between 5 to 71% depending on the setting where these studies have been performed. AxSpA prevalence is 5–24% in primary care [2–4] and 32–71% in secondary care [5–8]. Previous research has shown that early diagnosis and treatment of axSpA leads to better treatment outcomes. [9,10] The time between disease onset and diagnosis of axSpA however, is estimated to be around 8–10 years. [9,11] This delay may cause disabilities, a reduced quality of life and affect work participation. [12] Therefore, early recognition of axSpA patients from all CLBP is crucial. [13,14] In most countries CLBP patients are first seen and managed by general practitioners (GPs) or physical therapists. Therefore, GPs should be able to recognize the ‘red flags’ for axSpA. Early recognition seems difficult in primary care (PC) since the prevalence of CLBP is high and GPs’ awareness of SpA features are low. Worldwide 19% of patients (age 20–59 years) suffer from CLBP. [15] As a result, several referral strategies have been developed to help physicians identify patients at risk for axSpA within these CLBP patients. [16–19] Most referral strategies however, were developed in secondary care patients and have not been externally validated. Moreover, the effect of implementing these algorithms on outcomes from a patient’s perspective are scarce, but is an essential step before implementing these algorithms as digital filters in the referral process of GPs. [20–21]

In this study we assessed the effect of implementing a referral algorithm in primary care on disability caused by CLBP by using the Case Finding Axial Spondyloarthritis (CaFaSpA) algorithm: a validated and easy to use, non-invasive algorithm for the PC setting. [3,4]

## Methods

### Study design

The IMPACT study followed a cluster randomized controlled trial design (trial registration number: NCT01944163, Clinicaltrials.gov), which was carried out in the PC setting in The Netherlands. Each cluster contained the GPs from a single PC practice and their included patients. [22]

This study was approved by the medical ethics committee of the Maasstad Hospital in Rotterdam, The Netherlands (Trial registration number: 201340).

#### Participants

Dutch rheumatologists, widely spread over The Netherlands (Rotterdam, Breda, Groningen and Nijmegen), were invited to participate. General practices in the surrounding areas of participating Dutch rheumatologists were invited to participate by letter or personally. The exclusion criterion for general practices was lack of usage of the International Classification of Primary Care (ICPC) coding system for their patients. [23]

Patients between 18–45 years who had current low back pain (LBP) for more than 12 weeks, and were registered by means of the ICPC code L03 (LBP without radiation) were invited to participate by a research assistant. Patients willing to participate signed an informed consent form and were contacted to check in- and exclusion criteria and to register the result of the CaFaSpA referral strategy for referral to a rheumatologist. Patients’ exclusion criteria were having a clear medical explanation for the back pain (e.g. trauma, hernia nuclei pulposi), being mentally incompetent or having insufficient understanding of the Dutch language (written). The recruitment period of patients was between 10 September 2014 and 6 November 2015. Depending on the recruitment date patients were followed for four months. Follow-up period ranged between 10 January 2015 and 6 March 2016.

#### Cluster randomization

The block randomization schedule was computer generated and conducted by an independent person, who was not involved in patient care. Randomization was stratified for the number of GPs working in the general practices (one or two vs. more than two) to ensure a similar number of patients in both groups. Patients and GPs were unblinded, because of the nature of the intervention.

#### Intervention

The intervention was the use of the digital CaFaSpA referral strategy. In the control group usual primary care was based on the Dutch guideline for LBP. [24] The CaFaSpA referral strategy consists of four parameters: inflammatory back pain (IBP), a positive family history of axSpA, a positive response to treatment with NSAID’s and a duration of back pain for more than 5 years. [3,4] IBP is a questionnaire and all other three variables are questions that a GP can apply when a CLBP patient visits their practice units. [4] If at least two out of four referral items are present, referral to a rheumatologist is advised in the intervention group (Table 1). In an external validation study, the CaFaSpA referral strategy had a sensitivity of 75% and specificity of 58%.

**Table 1:** The CaFaSpA referral strategy.

Positive ASAS IBP questionnaire
Positive family history for spondyloarthritis
Good reaction to NSAIDs
LBP > 5years
If at least two out of the four referral parameters are present a referral to the rheumatologist is advised.

In the usual care group, GPs took care of their patients as usual. Due to ethical reasons the score of the CaFaSpA referral strategy was given to both the GP and patient at the end of the 4 months follow-up period.

#### Outcome measures

Our primary outcome was disability caused by CLBP, measured with the Roland Morris Disability Questionnaire (RMDQ) at baseline and 4 months. In the developmental phase of this study the RMDQ is regarded as a clinically relevant outcome measure for low back pain patients and used in clinical trials within this population.

The RMDQ consists of 24 statements about disability caused by LBP and has a scale of 0 to 24. [25] A higher score indicates a more severe disability. [26] The RMDQ was captured via questionnaires that were sent by email or post.

Secondary outcome was axSpA diagnosis made by a rheumatologist in the intervention group. The number of referrals to a rheumatologist was also assessed. Rheumatologists in this study performed their usual daily clinical practice. If patients did not seek a rheumatologist, despite of our referral advice, we registered the reasons for not visiting the rheumatologist as much as possible.

#### Sample size

For the power calculation we assumed a minimal clinical difference of 2.5 points in the RMDQ score after 4 months. [27–29] A value of 6.0 was assumed for the standard deviation, as found in the previous CaFaSpA study. [4] Without clustering, we estimated 180 patients (90 per arm) would be required to detect a minimal difference of 2.5 RMDQ; with 80% power, using a 2-sided 2 sample t-test at a 0.05 significance level. The effect of the referral strategy can only be assessed in patients with a positive result of the referral strategy. As in the previous CaFaSpA studies about 50% of patients scored positive on the referral strategy [3–4], a total number of 180 patients per arm would be required. Initially, an average cluster size of 16 patients was expected, while the intra-cluster correlation coefficient was assumed to be 0.05. [30] Hence, to account for clustering, the design effect was calculated as 1 + (16–1) * 0.05 = 1.75. Multiplying 180 patients per arm by 1.75 implied that a total number of 315 patients per arm should be included. When also assuming a lost-to-follow-up rate of 25% a total number of 840 patients (420 per arm) was initially calculated as the target for inclusion. [22] However, during the inclusion period of the study the average cluster size [7] was found to be substantially smaller than was initially expected [16]. Therefore a small adjustment to the required sample size was applied. The new design effect was recalculated as 1 + (7–1) * 0.05 = 1.3. This yielded a total sample size of 624 patients after applying the updated design effect and accounting for 25% lost-to-follow-up.

### Statistical analysis

STATA/SE 14.2 was used for data analyses. Descriptive statistics were performed to describe the baseline characteristics. The difference over time between the two groups were analyzed by a linear mixed-effects model using maximum likelihood estimation. Fixed effects included allocation group, result of the referral strategy (positive or negative referral strategy) and their interaction. A random intercept was included for general practice to take clustered randomization into account. This random intercept stand for the effect of different PC practices (i.e. clusters). This random intercept parameter can be interpreted as the variance of the deviances of the cluster-specific intercepts to the overall mean (the intercept estimated in the fixed effects). Hence one random intercept term may account for an arbitrary number of clusters. Repeated measures within patients (outcome measured at baseline and after 4 months) were modeled by an unstructured covariance structure.

The linear mixed model allows for patients to have a missing outcome at either baseline visit or after 4 months (but not all (both) visits) and yields unbiased estimates. A sub-analysis within the intervention group was performed to investigate the effect of our model on RMDQ scores in patients who received a positive or negative referral advice. Finally, a sensitivity analysis was solely performed within patients who responded positively to NSAIDs in order to examine a potential effect among the intervention and usual care group. In all analyses, a p-value < 0.05 was considered statistically significant.

## Results

**Fig 1:**
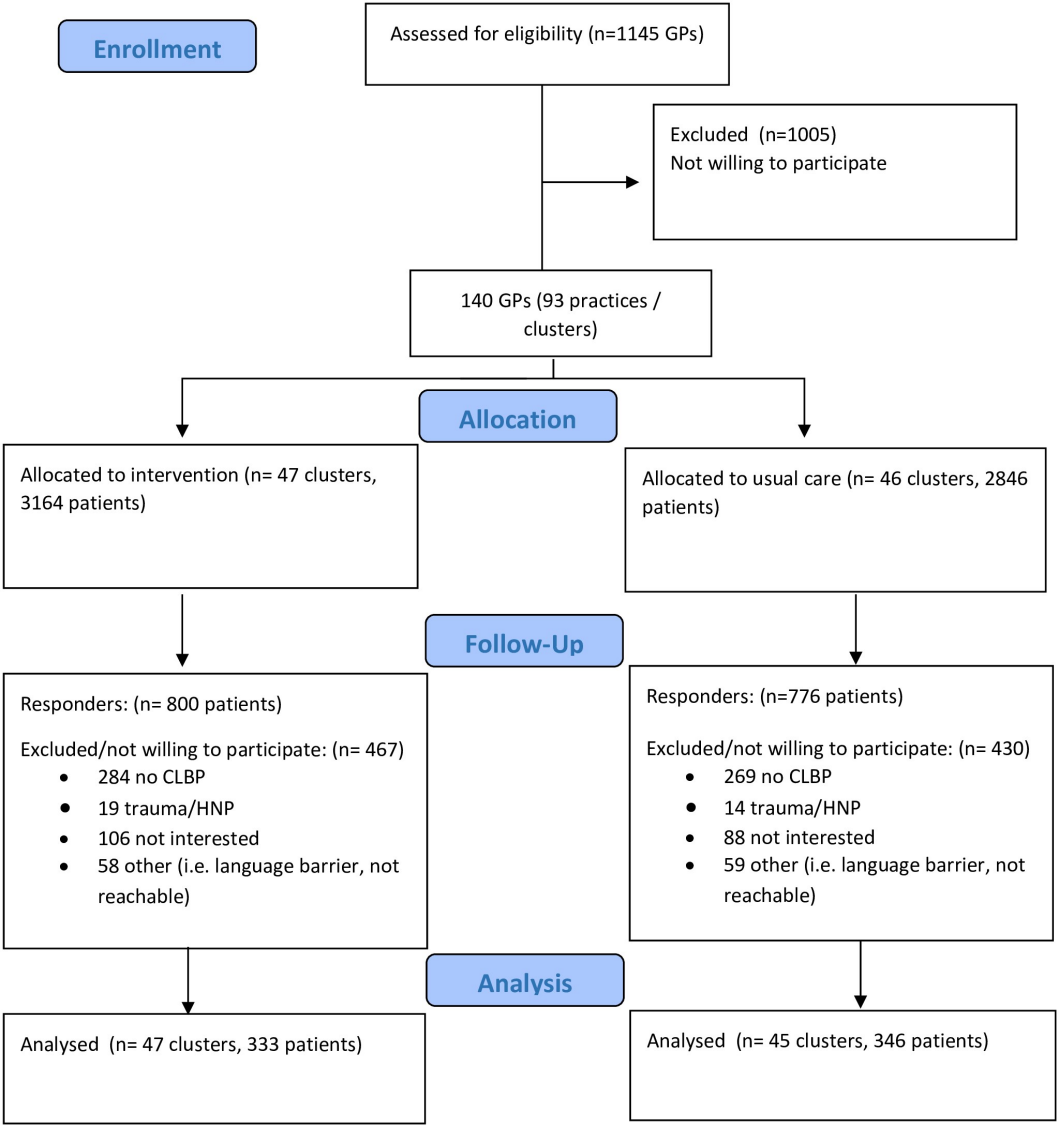
Recruitment flowchart impact study.

A total of 140 GPs (93 general practices) out of 1145 invited GPs were willing to participate (Fig 1). Following randomization of these 93 clusters, 47 general practices were assigned to the intervention group and 46 to the control group. Within these 93 clusters a total of 6010 patients were invited to participate, and 1576 responded to our invitation (intervention group n = 800 patients (25%), control group n = 776 patients (27%)). After checking the inclusion criteria by a research assistant, informed consent was obtained from 333 patients in the intervention group and from 346 patients in the control group. One cluster in the usual care group fell out because patients were either [1] not willing to participate [2] has no CLBP, [3] has trauma/HNP, [4] language barrier. This has led to a cluster size of 45 in the usual care group and a total of 679 patients and finally 92 clusters. The overall mean cluster size was 7.4 patients (SD 5.2).

### Baseline characteristics

The baseline characteristics of patients are shown in Table 2. Overall, our study population consisted of 64% women. The mean age was 36.2 years (SD 7.5) and the median duration of LBP was 10 years (interquartile range (IQR) 4–15 years). Approximately sixty percent of the patients had a positive outcome of the CaFaSpA referral strategy. The median RMDQ score at baseline was 8 (IQR 4–12) in both groups. In a sensitivity analysis we checked whether positive NSAIDs responders could have influenced our results. However, from our comparative analyses in baseline characteristics including age, gender, LBP duration, and NSAIDs use and dosage no significant differences were present between the intervention and usual care group (data not shown).

**Table 2:** Baseline patient characteristics.

	Use of referral strategy (n = 333)	Usual care (n = 346)
Number of clusters	47	45
Cluster size, mean ± SD	7.1 ± 4.9	7.7 ± 5.5
Age, year mean ± SD	36.7 ± 7.1	35.8 ± 7.8
Male sex, n (%)	115 (35)	130 (38)
CLBP duration, year median (IQR)	10 (4–15)	9 (4–15)
RMDQ, median (IQR)	8 (4–12)	8 (4–12)
VAS pain, median (IQR)	5 (3–7)	6 (3–7)
QoL mean ± SD	0.69 ± 0.26	0.70 ± 0.26
NSAID use, n (%)	88 (53)	87 (49)
*Individual components of referral model*		
Inflammatory back pain, n (%)	115 (35)	128 (37)
Positive family history, n (%)	82 (25)	71 (21)
Positive response to NSAIDs*, n (%)	154 (46)	192 (55)
CLBP ≥ 5 years	233 (70)	249 (72)
Positive referral model, n (%)	192 (58)	216 (62)

LBP: low back pain. CLBP: chronic low back pain. IQR: interquartile range. RMDQ: Roland Morris Disability Questionnaire. VAS: visual analog scale. Cluster size = number of patients. QoL: Quality of life measured with the EQ-5D.

*Positive NSAIDs response according to patients.

### Primary endpoint

In total, 577 patients (85%) completed the RMDQ at baseline and 484 (84%) patients completed the RMDQ after 4 months. At baseline the mean RMDQ score was 8.39 (7.59–9.18) in the intervention group and 8.61 (7.83–9.39) in the control group. At four months the mean RMDQ score for the intervention and control group was 7.65 (6.79–8.50) and 8.15 (7.34–8.96) respectively.

**Fig 2:**
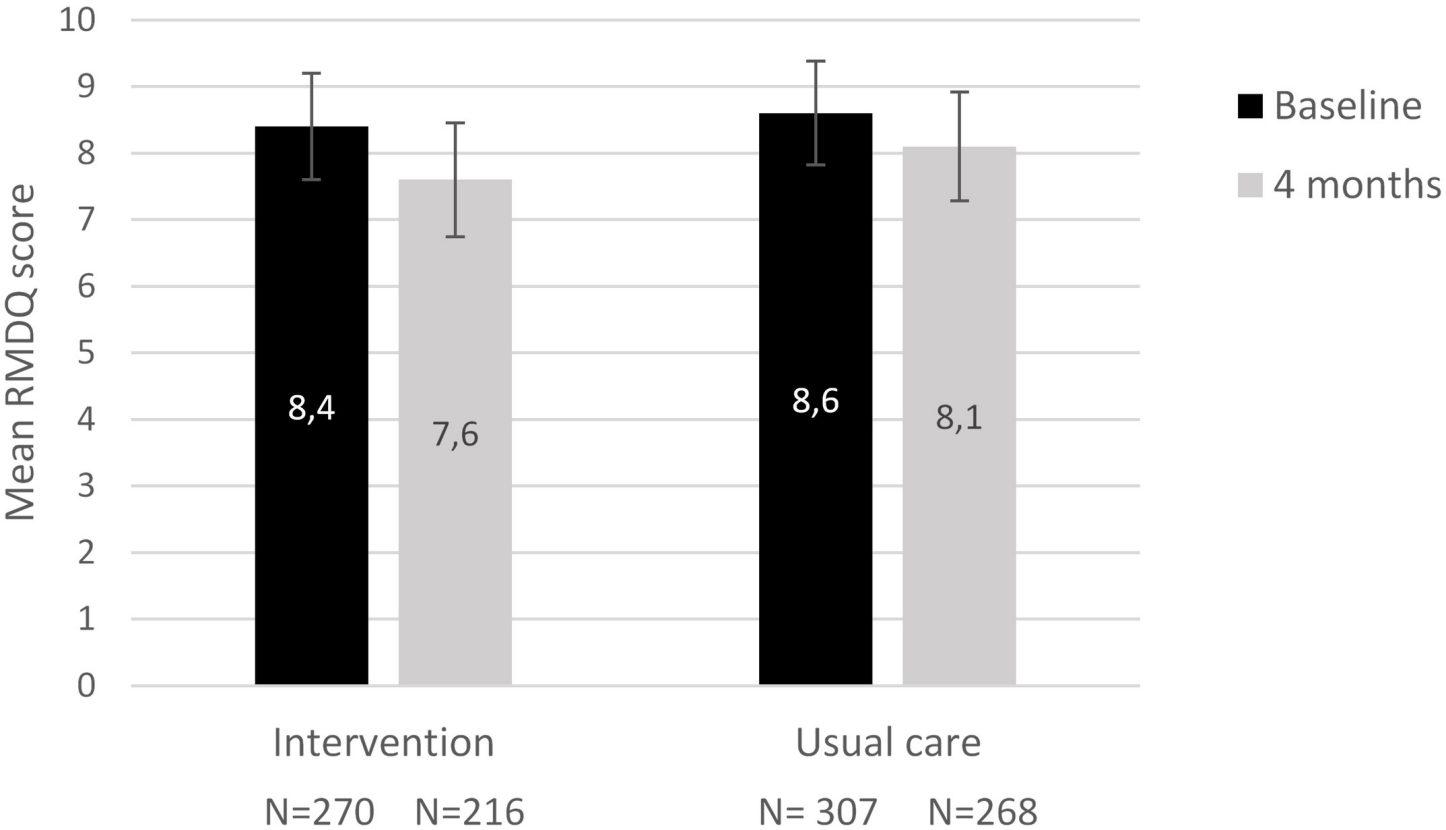
Estimated mean RMDQ scores over time for the overall intervention and usual care group. Bars indicate 95% confidence intervals for the mean estimates.

**Fig 3:**
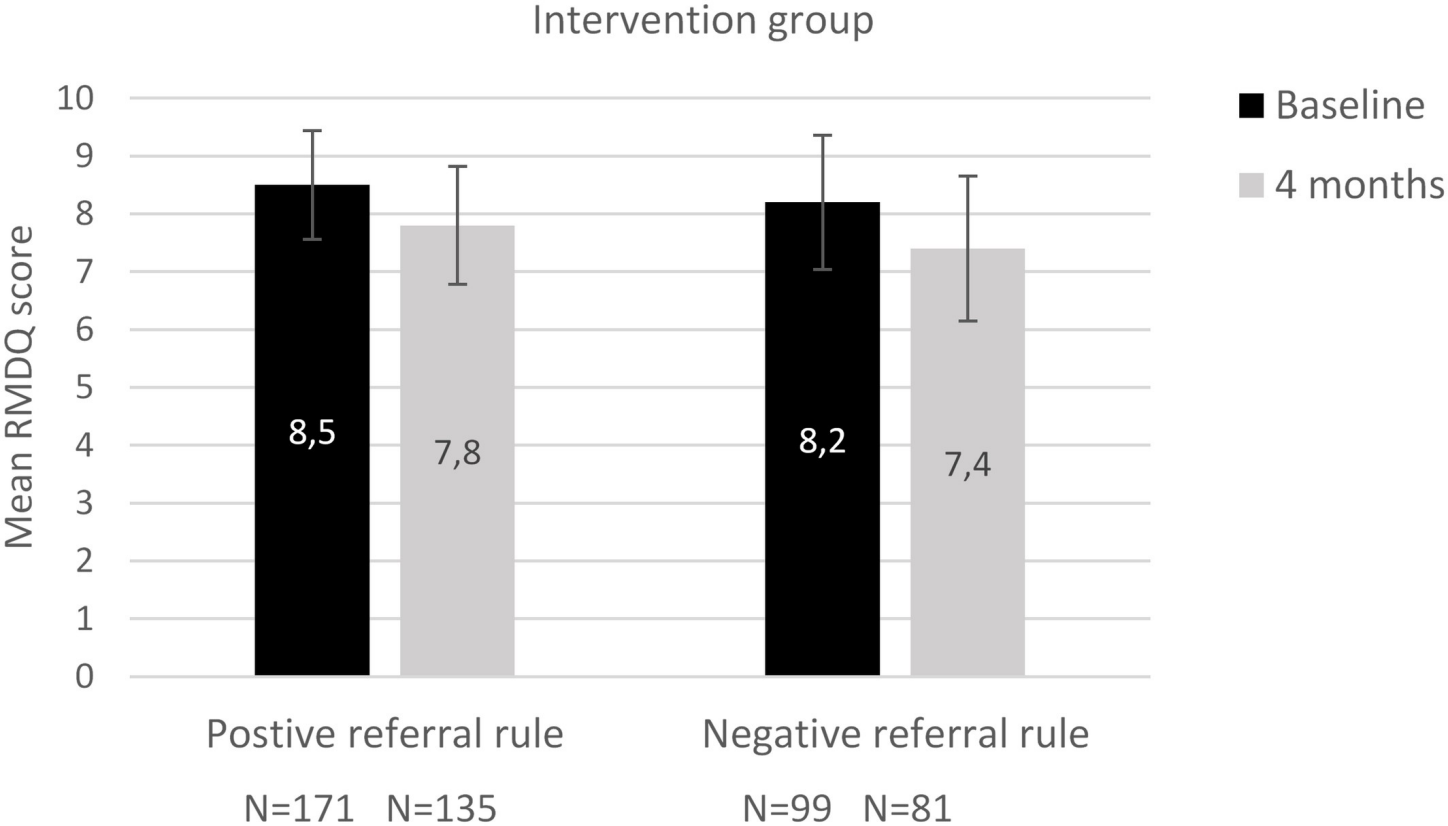
Difference in mean RMDQ scores over time within the intervention group, for patients receiving positive and negative referral strategy. Bars indicate 95% confidence intervals for the mean estimates.

A linear mixed-effects regression model was performed on 597 individual patients (47% intervention, 53% control), with at least one available RMDQ score (Fig 2). The mean difference of 0.28 between the groups was not statistically significant (p-value 0.50). Fig 3 shows the sub-analysis of the intervention group. The absolute mean decreases in RMDQ scores between the patients who received either a positive or negative referral advice was similar.

### Secondary endpoint

**Fig 4:**
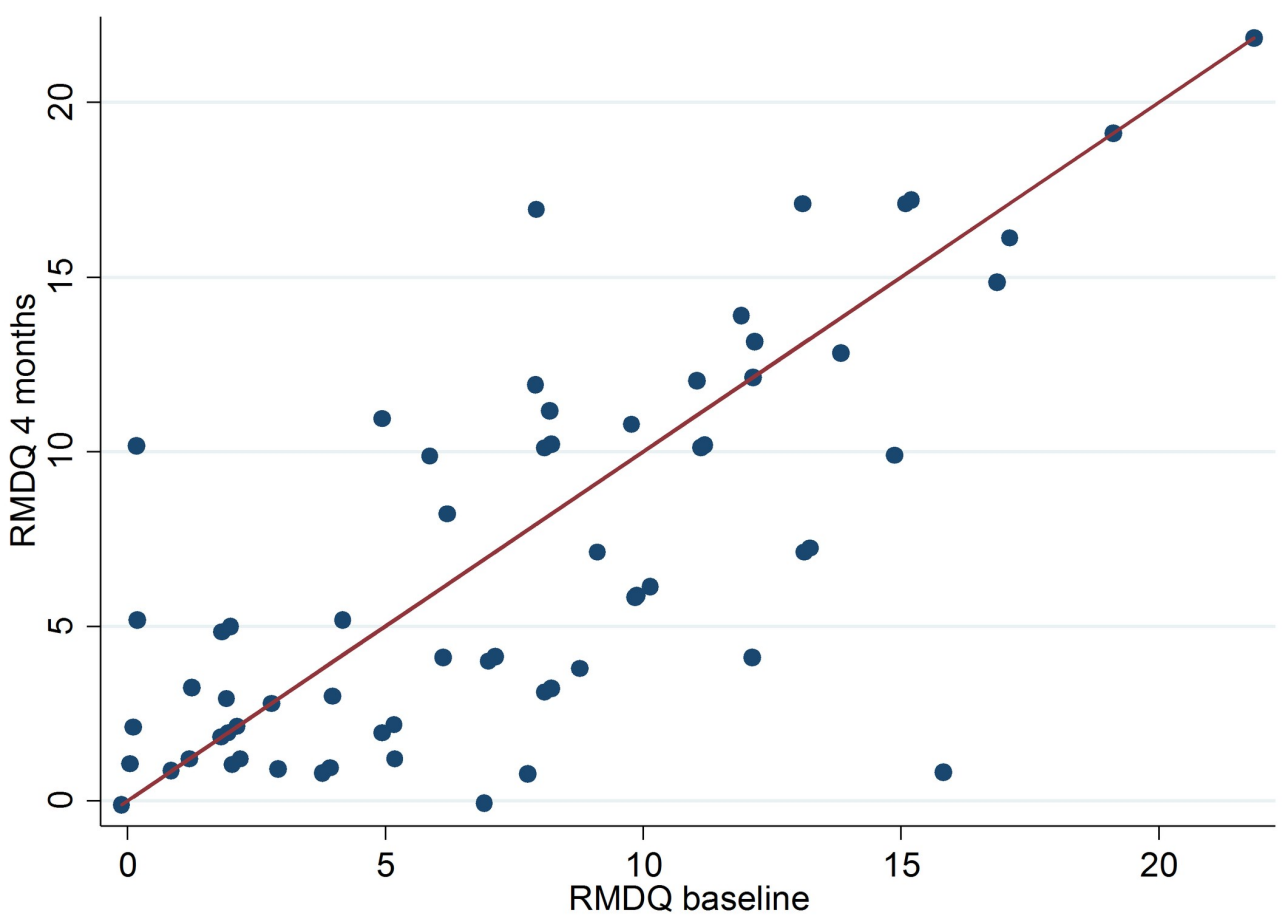
Scatter plot of the RMDQ scores at baseline and after 4 months in the intervention group in patients with a positive referral advice.

In total, 192 (58%) of the 333 patients in the intervention group received a positive referral advice based on the CaFaSpA rule. Of those finally 103 patients (54%) visited a rheumatologist. Out of the 103 patients we could only verify visits of 75 (73%) patients by receiving their hospital records. Six patients out of these 75 (8%) received the diagnosis axSpA from the rheumatologist. Among those patients one patient was treated with anti-TNF (Humira) and five patients received NSAIDs. The median RMDQ score among patients who visited the rheumatologist decreased from 8 to 5 after four months (p-value 0.17) (Fig 4).

## Discussion

In a previous prospective study the CaFaSpA referral strategy showed to be potentially efficient and discriminative for the identification of axSpA patients in a CLBP population. Therefore, we performed the current impact analysis, which is an essential step before implementation in daily practice. In this clustered randomized study, the CaFaSpA referral strategy did not have an effect on disabilities caused by CLBP compared to usual primary care after 4 months follow-up period. Although a small decrease in RMDQ scores was detected after 4 months, none of the patient groups reached a clinically meaningful decrease in RMDQ score of 2.5–5 points as described by previous studies. [27,28] To our knowledge this is the first study that examined the effect of a referral strategy for CLBP and for axSpA in daily clinical practice.

The lack of differences between the intervention and usual care groups might have been induced by a considerable short follow-up time as detectable treatment effects may take longer than 4 months. The first step in treatment is using at least two types of highly dosed NSAIDs for at least 4 weeks. When both treatments fail then anti-TNF alpha can be considered. It is expected that the difference between the two groups will be more obvious after a longer follow-up period of 12 months. In addition, we may have created awareness amongst GPs for axSpA or LBP complaints, even in the usual care group. Patients in the usual care group could have possibly received education, physiotherapy and advice in lifestyle to improve their CLBP, which might have positively influenced their RMDQ score.

Fortunately, the CaFaSpA referral strategy was able to identify newly diagnosed axSpA patients (8%), who had otherwise never been diagnosed and treated as described according to the international guideline. [31] This percentage is comparable to the minimally reported prevalence of axSpA among CLBP patients. [2] The lack of overall difference between the referred and usual care group might have been induced by the low prevalence of axSpA. The axSpA diagnosis in our study is lower than in the previously reported CaFaSpA studies (16% and 24%). [3,4]

In this study axSpA diagnose was made by a rheumatologist which reflects daily clinical practice. Currently we only have a classification criteria (ASAS) for axSpA and diagnostic criteria are still lacking.

The present study has some strengths and limitations. The strengths of our study are multifold. First, an impact analysis of a referral strategy for axSpA in PC has not been performed previously. Secondly, the design of this study as a clustered randomized trial is considered as the most suitable design to address this research question. Thirdly, we were able to include clusters with an equal number of participating patients in both groups (intervention and usual care). The statistical analyses, by using a linear mixed-effects regression model, take the cluster randomized nature of the study into account and is able to handle missing outcomes. Fourthly, in the present study we investigated the impact of the CaFaSpA referral strategy by means of patient relevant health outcomes (disability caused by CLBP). Overall, by using the CaFaSpA referral strategy, 42% of patients received a negative referral advice, who would otherwise be seen by a rheumatologist when the ASAS recommendation for CLBP was followed. [16] The ASAS referral strategy recommends that all CLBP patients with one axSpA feature should be referred to the rheumatologist. This would mean that almost all CLBP patients should be referred to a rheumatologist. Therefore, the CaFaSpA model can be used as an easy to use, non-expensive screening model in PC to identify young CLBP patients at risk for axSpA.

Finely, results of this study are generalizable since our baseline characteristics (including age, gender and LBP duration) and RMDQ scores are comparable with other Dutch studies in PC setting in patients with CLBP, where scores between 6 and 7 have been reported. [4,29]

Some limitations must also be addressed. First, NSAIDs use at baseline may have affected our estimates as NSAID’s are an over the counter medication in The Netherlands. However, our sensitivity analysis did not reveal any difference with regard to patient characteristics or clinically relevant parameters in those who had a good response to NSAIDs. Second, decisions not to seek a rheumatologist were made both at the patient’s and GP level. Despite fulfillment of the CaFaSpA referral advise, 39% of the patients either chose not to visit the rheumatologist due to financial reasons or because they personally did not suspect that their back pain was caused by axSpA. On the other hand, patients were advised by their GP not to seek the rheumatologist because the GP does not suspect an axSpA diagnose. Moreover, in those who had been referred to rheumatologist, the advised diagnostic workup of axSpA was not fully followed. This approach could have influenced our results. For example, only 89% of the patients had a conventional X-ray of the sacroiliac joints and in all patients at least two features were present. Therefore, HLA-B27 positivity or sacroiliitis on MRI should have been tested. [32] Finally, we want to highlight that the expected changes of disability would be much higher if treatment with TNF blockers would have been started.

However, four months follow-up is too short period for a patient to visit a rheumatologist, fail on two different NSAIDs and start biologicals.

In conclusion, the CaFaSpA referral strategy for axSpA did not have an impact on disability after four months caused by CLBP. However, it might still be used as a screening model for primary care to identify CLBP patients at risk for axSpA. We finally want to emphasize that impact studies on outcomes that really matter to patients should be performed before implementing these referral models in daily practice.

## Supporting information

S1 Dataset A wide version of the dataset used to reach conclusions. (DTA)

S2 Dataset A long version of the dataset used to reach conclusions. (DTA)

S1 Syntax Syntax used to obtain results of this study. (DO)

S1 CONSORT checklist CONSORT 2010 checklist of information to include when reporting a randomised trial*. (DOC)

S1 Study protocol (DOCX)
